# Antiatherosclerotic Effect of *Canarium odontophyllum* Miq. Fruit Parts in Rabbits Fed High Cholesterol Diet

**DOI:** 10.1155/2012/838604

**Published:** 2012-07-01

**Authors:** Faridah Hanim Shakirin, Azrina Azlan, Amin Ismail, Zulkhairi Amom, Lau Cheng Yuon

**Affiliations:** ^1^Department of Biochemistry, Faculty of Biotechnology and Biomolecular Sciences, Universiti Putra Malaysia, Selangor, 43400 Serdang, Malaysia; ^2^Department of Nutrition and Dietetics, Faculty of Medicine and Health Sciences, Universiti Putra Malaysia, Selangor, 43400 Serdang, Malaysia; ^3^Laboratory of Halal Science Research, Halal Products Research Institute, Universiti Putra Malaysia, Selangor, 43400 Serdang, Malaysia; ^4^Department of Basic Sciences, Faculty of Health Sciences, Universiti Teknologi Mara, Kampus Puncak Alam, 42300 Selangor, Malaysia; ^5^Department of Agriculture, Agriculture Research Centre, Semongok, Sarawak, 93720 Kuching, Malaysia

## Abstract

The effect of *C. odontophyllum* (CO) fruit parts was investigated in hypercholesterolemic rabbits. Forty-nine rabbits, which were randomly divided into seven groups of seven animals (*n* = 7), received a diet containing different parts of CO fruit parts for 8 weeks. The groups were as follows: (1) normal diet: NC group and (2) hypercholesterolemic diet: PC, HS (10 mg/kg/day simvastatin), HPO (20 g kg^−1^ oil extracted from the pulp of CO), HKO (20 g kg^−1^ oil extracted from the kernel of CO), HF (50 g kg^−1^ fullfat pulp of CO), and HD (50 g kg^−1^ defatted pulp of CO). Among these groups, rabbits receiving defatted pulp of CO showed the greatest cholesterol lowering effect as it had reduced plasma LDL-C, TC, and thiobarbiturate reactive substance (TBARS) levels as well as atherosclerotic plaques. The presence of high dietary fiber and antioxidants activity are potential factors contributing to the cholesterol lowering effect. Consequently, these results indicate the potential use of CO defatted pulp as a cholesterol lowering and antioxidant agent.

## 1. Introduction

Hypercholesterolemia is a condition that caused by overproduction of oxygen free radicals (OFR) and leads to oxidative stress [1]. Oxidative stress is defined as an imbalance between oxidants and antioxidants in favor of oxidants, thus potentially leading to damage to biological system [2]. OFRs have been implicated in the progression of atherosclerosis.

Increased in plasma LDL has been correlated with susceptibility to developing atherosclerosis. A high concentration of LDL can be attributed to the development of oxidized LDL (oxLDL) in hypercholesterolemia. It has been demonstrated that higher level of circulating oxLDL is found in patients with coronary heart disease (CHD) [3]. HDLs, however, have been shown to exhibit direct antiatherogenic properties in a wide array of animal studies [4, 5]. The counteract effect of HDL is due to reverse cholesterol transport that removes cholesterol from peripheral tissues [6].

Hypercholesterolemic atherosclerosis is always correlated with an increase in the lipid peroxidation product, malondialdehyde (MDA), which indicates high OFRs level, and a decrease in the antioxidant reserve [1]. Higher plasma levels of MDA have been found in atherosclerotic patients [7]. A reduction in atherosclerosis by antioxidants has been associated with a decrease in MDA [8] and an increase in the antioxidant reserve [9]. Thus, the search for compounds from nutraceuticals sources to reduce serum cholesterol and hypercholesterolemic atherosclerosis is crucial.

*Canarium odontophyllum* Miq. (CO) or dabai is a fruit belongs to Burseraceae family which consists of 100 species well distributed throughout tropical Africa, Asia, and the Pacific island [10, 11]. The fruit is oval in shape [12], rich source of protein, fat, carbohydrates, and minerals (sodium, calcium, and iron) [13]. The fat content is similar to that of olive and avocado fruits [14, 15]. The matured CO fruit is dark purplish. In Malaysia, CO has been classified as underutilized fruit and is listed as a plant genetic resources for food and agriculture in Sabah [11]. Since 1985, the Department of Agriculture (DOA) has performed number of studies on the collection, documentation, conservation, and improvement of this fruit [10]. Physicochemical characteristics showed that the fruit has high respiration production rate due to a short shelf life. Hence, the fruit must be handled properly and avoid noncold chain handling practice [12].

Considerable research has been performed worldwide on various fruits and fruit extracts for the prevention of coronary heart disease (CHD). Dietary antioxidants appear to play a protective role against the development of cardiovascular disease (CVD). The high antioxidant activity in CO fruit is a major aspect of this study. CO fruit has high antioxidant activity, determined *in vitro* using the DPPH scavenger activity, FRAP, and beta carotene bleaching assays [16, 17]. A study by Chew et al. [18] showed that CO fruit contains appreciable levels of phenolic compounds such as catechin, epigallocatechin gallate, and epicatechin.

The aim of this study was to investigate the effects of different parts of CO (fullfat pulp, defatted pulp, pulp oil, and kernel oil) in high-cholesterol diet fed rabbits on plasma lipids and plasma MDA. Histomorphometric intimal lesion analysis of the aorta was also investigated. The atherogenic index (TC to HDL-C ratio) was also calculated to determine the relative risk of CVD.

## 2. Materials and Methods

### 2.1. Sample and Preparation of Powders

Fresh fruits of CO were obtained from the Agriculture Research Centre, Semongok, Sarawak, Malaysia. The fruits were packed in an ice box (−4°C), transported to Peninsular Malaysia by air plane, and immediately delivered to Universiti Putra Malaysia. On arrival, the fruits without any physical damage were selected. The pulp was separated by peeling it off from pit (the inner part of fruit). The kernel was obtained by crushing the pit. Then, the pulp and kernel were freeze-dried (Virtis 5L, New York USA). After freeze-drying, the dried samples were ground using a dry grinder (Braun Multiquick ZK100 Germany) and sieved to obtain homogenized particles before undergoing oil extraction. The fatty acid composition of pulp and kernel oils obtained from the extraction was determined. Meanwhile, the proximate composition of full fat and defatted pulp was also determined.

### 2.2. Reagent

Chloroform, methanol, absolute alcohol, and 10% formalin were purchased from Merck (Germany), Cholesterol and Sudan IV were purchased from Sigma Chemical Co. (USA) and simvastatin was purchased from Pharmaniaga Logistic (Malaysia), and total cholesterol (TC), low density lipoprotein (LDL), high density lipoprotein (HDL), and triglycerides (TG) kits were from Roche Diagnostic (Germany).

### 2.3. Animals and Diets

Forty-nine male New Zealand white rabbits weighing 1.5–1.7 kg 8–10 weeks old were purchased from the East Asia Company, Malaysia. The animals were placed in individual cages at 25°C under a 12 h light/dark cycle. Water was provided *ad libitum*. Food intake was measured daily. Body weight was measured at baseline, at week 4 and at week 8. After two weeks of acclimatization, the rabbits were divided into seven groups of seven rabbits each (*n* = 7). Rabbits in group 1 were assigned as the negative control and consumed a normal diet (NC); group 2 was the positive control which consumed a 0.5% cholesterol-enriched diet (PC); groups 3 (HS), 4 (HPO), 5 (HKO), 6 (HF), and 7 (HD) were the supplemented groups which consumed cholesterol supplemented with simvastatin (10 mg/kg/day), pulp oil (20 g kg^−1^), kernel oil (20 g kg^−1^), fullfat pulp powder (50 g kg^−1^), or defatted pulp powder (50 g kg^−1^ of CO fruit), respectively (Table 1). All groups received experimental diet prepared within three days before feeding.

### 2.4. Experimental Protocol

Simvastatin, pulp, and kernel oils were orally given in groups 3 (HS), 4 (HPO), and 5 (HKO), respectively. The simvastatin was diluted in 2 mL of distilled water. Meanwhile, the fullfat pulp and defatted powder received by groups 6 (HF) and 7 (HD) were included in the food pellet.

## 3. Methods

### 3.1. Sample Preparation

Pulp and kernel oils of CO were obtained by means of solvent extraction. Dried, homogenized, and ground pulp powder was soaked in chloroform-methanol (2 : 1 v/v) at ratio of 1 : 5 (w/v). A mixture of the sample and the solvent was kept overnight at room temperature and filtered the next day. The organic solvent was completely evaporated using a rotary evaporator (Buchi Rotorvapor R-200, Berlin, Germany) at 40°C. The residue was resoaked with fresh solvent twice to ensure complete extraction of the oil. The extracted oil was combined, weighed, and stored at −20°C until further analysis. The residues from the pulp were dried in an oven (Memmert, Schwabach, Germany) at 40°C to obtain defatted pulp. The contents of dried kernels were prepared in the same manner to obtain kernel oil.

### 3.2. Determination of Fatty Acid Composition

The fatty acid composition of the oil which was extracted using a chloroform-methanol mixture was determined by gas chromatography (Hewlett-Packard, HP1100, USA Agilent Technology). Fatty acid methyl esters (FAMEs) were prepared by weighing out 100 mg of the sample in a 20 mL test tube (with a screw cap). Then, the sample was dissolved in 10 mL of hexane. Next, 100 *μ*L of 2 N potassium hydroxide in methanol was added. After vortexing (BOECO, Germany) for 30 seconds, the mixture was centrifuged. Next, 2 mL of the clear supernatant was transferred to a sample vial for fatty acid composition analysis.

### 3.3. Gas Chromatography (GC) Analysis

The fatty acid composition of the oil samples was analyzed using an Agilent 6890 GC (USA Agilent Technology) equipped with split-splitness injector, a detector, and a Hewlett-Packard EL-980 flame ionization detection (FID) system to separate and quantify each FAME component. FAMEs were separated using a DB-23 column (60 m × 0.25 mm I.D., 0.15 *μ*m polyethylene glycol film). Chromatography data were recorded and integrated using Chemstations software (Version 6.0). The oven temperature was held at 50°C for 1 min, then increased to 175°C at 4°C/min and lastly finally increased to 230°C, holding for 5 min at 230°C. The temperatures for the injector and detector were set at 250°C and 280°C, respectively. One microliter of the sample volume was injected with a split ratio of 1 : 50 at a column temperature the 110°C. Carrier gases that were used in the system were helium gas, 1.0 mL/min controlled at 103.4 kPa, the hydrogen and air used for FID were held at 275.6 kPa.

### 3.4. Preparation of Diets

The experimental diets, as shown in Table 2, were prepared based on the NC diet, which is a normal basal diet. The normal basal diet contains soybean (150 g kg^−1^), corn (30 g kg^−1^), starch (100 g kg^−1^), molasses (20 g kg^−1^), corn oil (20 g kg^−1^), a vitamin mixture (3 g kg^−1^), a mineral mixture (3.5 g kg^−1^), DL-methionine (2 g kg^−1^), calcium carbonate (5 g kg^−1^), and calcium hydrogen phosphate (5 g kg^−1^).

The basal diet for the NC group was prepared as follows: soybean, corn, and palm kernel cake were ground using an electric grinder, weighed and mixed manually. Later, the minerals and vitamin mixtures (minerals mixture, vitamins mixture, CaCO_3_, CaHPO_4_, and DL-methionine) were added. Next, oil and molasses were mixed. Starch (100 g kg^−1^) was added to the mixture, mixed well, and placed carefully on a dish covered with aluminum foil. Then, the dough was cut into small pieces and dried in an oven (Memmert, Schwabach, Germany) at 45–50°C overnight. The prepared pellets were stored in air-tight containers at room temperature. For the hypercholesterolemic diets (HS, HPO, HKO, HF, and HD), cholesterol (5 g kg^−1^) was added.

Similarly, the hypercholesterolemic diets were prepared using the same method as described for the NC diet with the addition of other food ingredients in the particular diet. In the HS group, a hypercholesterol diet was given, with simvastatin (10 mg/kg per day) given orally by force-feeding. The simvastatin was prepared by dissolving simvastatin with distilled water. For the HPO and HKO diets, 20 g kg^−1^ of pulp (PO) and kernel oils (KO) extracted from the pulp and kernel of CO were added, respectively, which replaced the total fat required for the rabbits. Meanwhile, for the HF diet, 50 g kg^−1^ of fullfat pulp was added to represent the replacement of total fat and 80 g kg^−1^ of carbohydrate required. For the HD diet, 50 g kg^−1^ of CO defatted pulp was added. Defatted pulp is a rich source of dietary fiber, thus representing 14% replacement of the daily requirement of dietary fiber in the HD diet. Previous studies have shown that giving 100–150 g kg^−1^ of dietary fiber from fruit such as defatted roselle [19] and defatted cocoa [20] has cholesterol-lowering effects in a hypercholesterolemic model. Thus, 50 g kg^−1^ of defatted pulp may potentially provide a similar effect.

### 3.5. Blood Sampling

 Blood was drawn from marginal ears of the rabbits at weeks 0 (baseline), 4, and 8 from all groups. The blood was collected into an EDTA tube. All experimental protocols were approved by the Animal Care and Use Committee (ACUC) of the Faculty of Medicine and Health Sciences, Universiti Putra Malaysia (UPM), Serdang, Selangor. Plasma was obtained by centrifugation at 3000 rpm for 10 minutes at 25°C. Plasma was used for determination of TC, LDL-C, HDL-C, TG, and MDA levels.

### 3.6. Body Weight and Food Intake

 Individual body weight and food intake of rabbits were recorded at week 0 and 8. 

### 3.7. Biochemical Analysis

Plasma lipids were estimated spectrophotometrically using an automatic chemistry analyzer (Hitachi, UK) with standard test kits (Roche Diagnostics, UK). The plasma TBARS level was measured using the thiobarbituric acid (TBA) assay described by Buege and Aust [21].

### 3.8. Determination of Atheroma Plaques in the Aorta

At the end of week 8, the rabbits were sacrificed. The aorta between its origin and bifurcation into the iliac arteries was dissected, opened longitudinally, and prepared for accurate detection and estimation of lipid deposits in the intima by the macroscopic method as described by Holman et al. [22]. The aortas were washed out with normal saline and used for Sudan IV staining.

After aortas were dissected and properly cleaned of fat residue on the outer surface, sectioned longitudinally, and stretched onto a piece of board followed by fixation in 10% formalin for 24 hours. Subsequently, they were rinsed off with 70% alcohol to remove the formalin residue. Then, the aortas were immersed in Herxheimers solution (Sudan IV in ethanol and acetate) for 2-3 minutes at room temperature. Sudan IV stain is lipophilic, so it will stain the lipid components on the surface of the tissue. After 15 min, aortas were consecutively washed in running tap water for 1 hour. Water flow was not too heavy to avoid damage to the tissues. This staining allows for a clear illustration of plaques due to their deep red color.

Images of the aortas were captured using a digital camera (EOS Canon, Japan). The total atherosclerotic areas of the intimal surface of the aorta were measured in mm^2^ using graph paper. The extent of atherosclerosis was expressed as the percentage of the luminal surface covered by atherosclerotic changes. The lesion area was estimated using the following formula described by Bocan et al. [23]:

(1) Lesion area=lesion area on⁡ intimal surface of the aortawhole area of the aorta.

### 3.9. Statistical Analysis

The data are presented as group means ± SEM. Analysis of variance (ANOVA) using Statistical Package Social Sciences (SPSS) version 16.0 was used for data analysis. Statistical differences between the treatment and control groups were determined using one-way ANOVA. The Turkey post-hoc test was used for multiple group comparisons. The significance value was set at *P* < 0.05.

## 4. Results and Discussion

### 4.1. Nutrient Composition of Fullfat and Defatted Pulp of CO Fruit

Table 1 shows the nutrient composition of fullfat and defatted pulp of CO. The fruits are a rich source of fat, dietary fiber, and carbohydrates. This finding is in accordance with a study by Hoe [13], who reported that CO is rich in fat and carbohydrates. However, the dietary fiber was lower compared to the present study which could be due to the different determination method used. The fat content in this study was slightly higher than the level reported by Hoe (260 g kg^−1^) [13]. The fat content of CO is lower compared to other species of *Canarium* [24]. The moisture, fat, and protein levels reported by Chew et al. [25] are higher than present finding because the values were based on fresh weight of fruit sample.

To our knowledge, this is the first time the value of total dietary fiber (TDF) of CO fruit has been reported. The TDF level in the fullfat pulp was two-times higher than in the defatted pulp. The insoluble dietary fiber (IDF) was higher than the soluble dietary fiber (SDF) in fullfat and defatted pulp powder. Besides, the dietary fiber of CO pulp was also higher than olive [26] but lower than other fruits such as persimmon [27], carob [28], and apple [29].

### 4.2. Fatty Acid Composition of Pulp and Kernel Oils of CO Fruit

Table 3 shows the fatty acid composition of pulp and kernel oils extracted from CO fruit. The fatty acids that were found abundantly in the studied oils were oleic acid (18:1), palmitic acid (16:0), and linoleic acid (18:2n6cis). Pulp oil share similar fatty acid composition with palm oil, in which equal SFAs and MUFAs content are found in these oils. Meanwhile slightly higher SFAs compared to UFAs were found in kernel oil. Similar fatty acid compositions of pulp oil have been reported by previous investigators [30] but with slightly higher PUFA (14.05% versus 12.76%). Furthermore, a similar fatty acid composition in pulp oil was reported by Azrina et al. [17].

### 4.3. Food Intake and Body Weight

The food intake and body weight in all groups were increased throughout the experimental period (Table 4). At the end of study, the highest body weight was found in the PC group. The increment might have been due to greater lipid deposition in the body tissue of this animal [31] in accordance with a previous study by Lee et al. [32]. Among the treated groups, the highest body weight was found in HD, followed by higher food intake. Higher food intake in the CO-treated group could have been due to high satiety. Thus, it can be suggested that this fruit is best consumed by humans. However, the lowest food intake in HS could have been due to low satiety and distaste for simvastatin [33].

### 4.4. Lipid Profile

Hyperlipidemia, especially hypercholesterolemia, is a major risk factor for coronary artery disease, and reducing plasma cholesterol levels in particular LDL cholesterol may reduce the risk of this disease. Globally, the morbidity of cardiovascular disease has increased slightly, making hyperlipidemia the leading cause of mortality. Meanwhile, epidemiologic studies have found that increased serum cholesterol predict the risk of CHD in various human and animal studies [34, 35]. Therefore, dietary strategies known to reduce cholesterol levels, such as a reduction in total and saturated fat intake as well as increased intake of dietary fiber [36], are meaningful steps to minimize the risk.

Based on data in Table 5, at baseline, the plasma TC, LDL-C, and HDL-C levels were not significantly different from each other in all groups as we had standardized the animals' condition, in terms of the room environment and fasting period [35]. For changes in lipid indices, the high cholesterol diet fed rabbits (PC) showed significantly (*P* < 0.05) increased in TC, LDL-C, HDL-C, and TG levels compared to normal animals (NC). The TC was increased 12-fold in the first four weeks, and continued to increase a further 1.8 times by week 8. This showed that hypercholesterolemia had been successfully established in the rabbits by means of feeding 5 g kg^−1^ cholesterol in the diet over four weeks. HDL exhibits antiatherogenic effects, but the level was higher in PC. This could be due to the cholesterol load on plasma lipids, which increases the work load of plasma lipoproteins especially HDL. In the present study, simvastatin was used because it is a potent hypolipidemic drug known to exert its action by inhibiting HMG-Coa reductase, the rate-limiting step in cholesterol biosynthesis. In humans, the statin family has been well accepted as cholesterol-lowering drugs [37]. Thus, the HS group was used to compare the effectiveness of the fruit parts in reducing plasma cholesterol levels. Based on the present data, the simvastatin treated group had significantly reduced (*P* < 0.05) TC, LDL-C, and TG levels, which are all in accordance with previous studies [37–39].

Among the treatment groups, rabbits fed with the defatted pulp of CO (HD) showed the greatest reduction in plasma TC, thus indicating a positive hypocholesterolemic effect (markedly reduced TC and LDL-C, *P* < 0.05) of the defatted pulp in the context of exposure to a high cholesterol diet. The superior effect of the HD diet could have been due to the 70 mg of polyphenolic compounds found in the defatted pulp as shown by a previous investigator [40]. A possible mechanism involved in its *in vivo* antioxidant effect may be mediated by radical scavenging activity [16]. The other possible mechanisms could be the effect of bile acid binding by polyphenols which in turn increase fecal loss [28]. The hypocholesterolemic effect of defatted pulp could also be due to the presence of carotenoid content especially *trans*-b-carotene, 15-*cis*-b-carotene, 9-*cis*-b-carotene, and 13-*cis*-b-carotenes. The peel fraction which represents about 30% from the defatted pulp exhibits good inhibitory effect against hydrogen peroxide-induced haemoglobin oxidation, ranging from 45.3 to 59.7% [41].

Defatted pulp of CO consists 50% dietary fiber enriched with 70 mg of polyphenol compounds and exhibited a cholesterol lowering effect. It is well known that dietary fiber in the presence of antioxidant compounds plays an important role in reducing plasma cholesterol levels. Many animal and human studies have shown that consumption of food rich in dietary fiber with the presence of antioxidant compounds has positive effects on different parameters associated with CVD such as endothelial function, platelet activation, and biomarkers of lipid peroxidation [20, 42, 43]. It is well known that dietary fiber plays an important role in reducing plasma cholesterol levels. Previous studies have revealed that fiber-rich food such as oat bran, beans, grains, and legumes have significant effects on serum cholesterol [42, 44, 45]. Various actions of dietary fiber have been shown to lower serum cholesterol, such as hindering digestion and absorption of dietary fat, modifying bile acid absorption and the metabolism and formation of short chain fatty acids [20, 46]. Moreover, numerous studies have demonstrated that the reduction of plasma cholesterol level is likely attributed to bile acid and dietary fat binding to the fiber compound [20, 47, 48]. Therefore, it can be proposed that the cholesterol lowering effect of defatted pulp in the HD group in the present study might have been due to the binding of dietary fibre in the defatted pulp with bile acids which in turn increases the faecal excretion [46, 47].

HDL-C plays an important role in protection against cardiovascular diseases and is responsible for transporting cholesterol from cells and arteries to the liver for catabolism. In the CO supplemented groups (HPO, HKO, HF, and HD), the increment of HDL-C was higher (6.4–9.5 times) than the statin receiving group (HS) (2.6 times). Thus, it can be suggested that the effect of CO fruit parts on lowering plasma lipid cholesterol is superior to that of simvastatin. Triglycerides (TGs) circulate in the blood are stored in the body fat and are used when the body needs extra energy. Excessive TG is related to the occurrence of coronary artery disease [49]. In this study, feeding rabbits a high cholesterol diet (PC) increased the plasma TG by two-fold from week 0 to week 8. Meanwhile, supplementation of fullfat pulp in rabbits resulted in 8% a significant decrease (*P* < 0.05) in TG after 8 weeks of treatment. No significant changes (*P* > 0.05) were observed in groups supplemented with pulp oil (HPO), kernel oil (HKO), and defatted pulp (HD), compared to PC.

Generally, it is difficult to detect the CVD risk factor based on individual lipoproteins (TC, LDL-C, HDL-C) and TG levels. Consequently, the atherogenic index (TC to HDL-C ratio) was used for this purpose. This ratio is a good marker for identifying and minimizing the risk of CVD [50]: an increase in this ratio increases the risk of cardiovascular diseases. The value for the TC to HDL-C ratio in the PC group was three times higher compared to animals in the normal group, which clearly showed that PC is associated with a very high risk of CVD. After treatment with simvastatin, this value decreased by two-fold. In the blood, statins lower TC, LDL-C, and TG. Interestingly, supplementation of both fullfat pulp and defatted pulp of CO decreased the ratio of TC to HDL by 36% and 93%, respectively, with no significant differences from the statin group.

Besides dietary content and other nutrients, the fatty acid content can influence plasma lipid and lipoprotein types and concentrations. Saturated fatty acids (SFAs) are well known to increase CVD by elevating plasma TC and LDL, while a diet rich in monounsaturated fatty acids (MUFAs) and polyunsaturated fatty acids (PUFAs) is able to reduce plasma TC and LDL in normolipidemic subjects [51]. The plasma TC was reduced by 28% and 30% in the HPO and HKO groups, respectively. The preventive roles of pulp and kernel oils of CO were attributed to their MUFA contents. A diet high in MUFA is able to suppress LDL oxidation and oxidative stress [52]. However, the plasma LDL-C levels were slightly higher in HKO. This could be explained by higher levels of SFAs in kernel oil. A study by Mensink et al. [51] showed that vegetable oils rich in SFAs can increase plasma LDL-C.

### 4.5. Lipid Peroxidation by Thiobarbituric Acid Reactive Substances (TBARS)

Lipid peroxidation can be evaluated by the thiobarbituric acid reactive substances method (TBARS) which evaluates oxidative stress by assaying for MDA (malondialdehyde), a product of lipid breakdown [9].  Table 6 shows the effect of CO fruit parts on the TBARS level at different time points where initial values of plasma TBARS were not significantly different to each other. In rabbits, a fed high cholesterol diet, the plasma TBARS was significantly higher (*P* < 0.05) than in rabbits on a normal diet, which clearly indicates elevated production of free radicals which enhance the process of lipid peroxidation [8].

The TBARS level was diminished significantly (*P* < 0.05) in groups supplemented with the fruit parts of CO. The reductions in the HPO, HKO, HF, and HD groups were 14%, 13%, 15%, and 36%, respectively; the highest reduction in TBARS was observed in the defatted pulp supplemented group. The reduction in the lipid peroxidation level could be related to strong antioxidant properties of the defatted pulp against OFRs in biological systems. CO fruit pulp has been demonstrated to have a high polyphenols contents with potent antioxidant capacity *in vitro *[16, 17]. The antifree radical activities of phenolics are well established, act as free radical scavengers and slow down oxidative stress-related lipid peroxidation.

### 4.6. Atheroma Plaques

**Figure 1:**
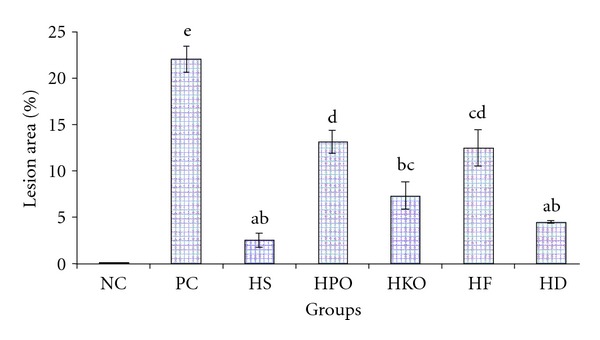
Percentage of lesion area in the experimental groups. Each value represents the mean ± SEM for *n* = 7. Values with different letters are significantly different (*P* < 0.05) between groups.

**Figure 2:**
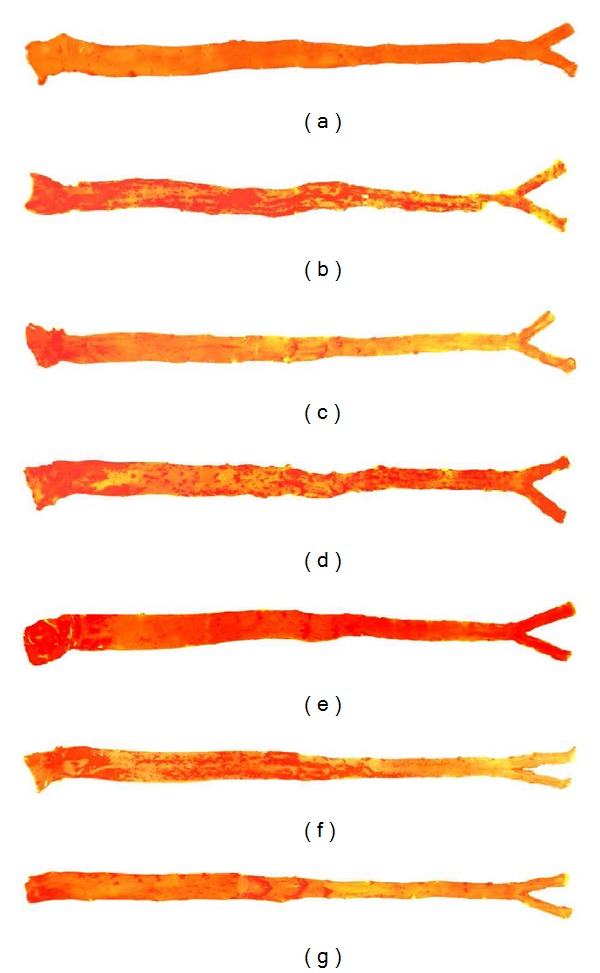
Photograph of intimal surface of aorta of each group. Representative photograph of intimal surface of aortas stained with Sudan IV. Atherosclerotic lesions were indicated by concentrated brick red colours. Athersclerotic lesion is absent in the NC group. (a) NC, (b) PC, (c) HS, (d) HPO, (e) HKO, (f) HF, (g) HD followed the respective diet for 8 weeks.

Atherosclerosis is characterized by the accumulation of cholesterol deposits in the macrophages of arteries [53] which become disrupted through the physical forces in arterial walls. The severity of atheromatous lesions is associated with hypercholesterolemia [54, 55]. The percentages of lesions on the intimal surface of aortas from the PC, HS, HPO, HKO, HF, and HD groups are shown in Figure 1. The plaques were severe in animals fed a high cholesterol diet (PC) (22.08% ± 2.76) (Figure 2). A hypercholesterolemic diet produced intimal thickening that contained foam cells similar to those observed by many researchers [54, 56, 57].

Among the treated groups, HD exhibited the greatest reduction in atherosclerotic plaque formation by nearly 80%. The pronounced effect in HD compared to HF could be due to the higher level of dietary fiber in defatted compared to fullfat pulp (Table 1). Pulp oil had higher MUFA compared to kernel oil. However, lower plaque formation was found in HKO compared to HPO which could be due to higher level of cholesterol-lowering agents such as phytosterols [58], antioxidant vitamins [59], and flavonoids [2] which were not measured in this study.

The protective effect of the defatted pulp could be attributed to the high antioxidant capacity of the fraction that reduced the plasma MDA level (Table 6). It has been well established that an increased MDA level is positively correlated with the progression of atherosclerosis [60]. The high polyphenolic contents of the defatted pulp [16, 40] used in the present study might have prevented the development of atherosclerosis by its free radical-scavenging antioxidant activity. Instead of having high antioxidant activity, the protective effect of the defatted pulp could also be attributed to its lipid lowering ability. Animals fed defatted pulp had reduced (significant, *P* < 0.05) plasma TC and a lower atherogenic index (TC to HDL-C ratio). Several epidemiological studies have demonstrated that increased dietary intake of natural phenolic antioxidants plays an essential role in the prevention of cardiovascular diseases, cancer, and neurodegenerative diseases. Similarly, the pulp of CO fruits has been shown to possess tremendous *in vitro* antioxidant capacity estimated from extracts of the fruit [16, 17, 40]. In addition, a high correlation was reported between the phenolic content and antioxidant capacities in these studies. Thus, this suggests that the protective effect of CO defatted pulp against the formation of atherosclerotic lesions may be mainly attributed to the high antioxidant content of this fruit part.

## 5. Conclusions

Among the CO fruit parts, defatted pulp was associated with the greatest reduction in atherosclerotic plaque formation, induced by a significant reduction in LDL-C, TC, and lipid peroxidation levels. The presence of high dietary fiber and high levels of antioxidants with potent activity was essential factor contributing to the retardation of atherosclerosis and a reduction in coronary artery disease risk. Consequently, these results indicate the potential use of CO defatted pulp as a hypocholesterolemic and antioxidative agent apart from its ability to slow the progression of atherosclerosis.

## Figures and Tables

**Table 1:** A proximate composition of fullfat and defatted powder of *C. odontophyllum* Miq.

Constituent	Percentage (g kg^−1^)	
	Fullfat pulp powder^1^	Defatted pulp powder^2^
Moisture^a^	73.4 ± 0.93	85.2 ± 0.06
Fat^a^	454.3 ± 0.15	ND
Protein^a^	67.1 ± 0.09	124.0 ± 0.07
Carbohydrate^a^	180.2 ± 0.08	140.6 ± 0.45
Ash^a^	37.0 ± 0.04	63.0 ± 0.05
TDF^b^	297.8 ± 2.00	545.6 ± 2.08
SDF^b^	79.6 ± 3.26	145.9 ± 3.61
IDF^b^	218.1 ± 0.04	399.7 ± 0.56

^a^Values are based on triplicate determinations.

^b^Values are based on duplicate determinations.

^1,2,3^Values are based on 100 g of fullfat and defatted pulp of *C. odontophyllum* fruit respectively,

TDF: total dietary fiber.

IDF: insoluble dietary fiber.

SDF: soluble dietary fiber.

ND: not detected.

**Table 2:** Formulation of experimental diets (g kg^−1^).

Ingredients	Experimental diets (g kg^−1^)						
	NC	PC	HS	HPO	HKO	HF	HD
Soybean meal	150	150	150	150	150	150	150
Corn	300	300	300	300	300	300	300
Palm kernel	360	360	360	360	360	330	310
Starch	100	100	100	100	100	100	100
Molasses	20	20	20	20	20	20	20
Corn oil	20	20	20	—	—	—	20
Vitamin mixture^a^	3	3	3	3	3	3	3
Mineral mixture^b^	35	35	35	35	35	35	35
DL-methionine	2	2	2	2	2	2	2
CaCO_3_	5	5	5	5	5	5	5
CaHPO_4_	5	5	5	5	5	5	5
Cholesterol	—	5	5	5	5	5	5
Pulp oil of CO	—	—	—	20	20	—	—
Kernel oil of CO	—	—	—	—	—	—	—
Fullfat of CO pulp	—	—	—	—	—	50	—
Defatted CO pulp	—	—	—	—	—	—	50

Total	1000	1005	1005	1005	1005	1005	1005

High cholesterol diet group; HS: treated with simvastatin, HP: treated with pulp oil of *C. odontophyllum*, HK: treated with kernel oil of *C. odontophyllum*, HF: treated with fullfat pulp of *C. odontophyllum*, HD: treated with defatted pulp of *C. odontophyllum.* ^a^Vitamin mixture: Vit A: 50000 i.u., Vit D3: 8000 i.u., Vit E: 8 mg. ^b^Mineral mixture: manganese: 320 mg, zinc: 200 mg, magnesium 1400 mg, iron: 300 mg, copper: 50 mg, cobalt: 10 mg, iodate: 20 mg, phosphorus: 10000 mg, sodium chloride: 5500 mg, and calcium: 1300 mg.

**Table 3:** Fatty acid composition of pulp and kernel oils of *C. odontophyllum* fruits.

Fatty acids	Percentage (%)	
	Pulp oil	Kernel oil
Saturated fatty acid	43.42 ± 0.05	56.2 ± 0.04
14:0	0.31 ± 0.04	0.1 ± 0.01
16:0	40.31 ± 0.01	50.22 ± 0.74
18:0	2.19 ± 1.04	5.88 ± 0.76
20:0	0.61 ± 0.07	ND
Monounsaturated fatty acid	42.53 ± 0.06	39.84 ± 0.04
16:01	0.63 ± 0.08	0.28 ± 0.05
18:01	41.9 ± 0.02	39.56 ± 0.48
Polyunsaturated fatty acid	14.05 ± 0.09	3.96 ± 0.04
18:2n6cis	14.05 ± 1.96	3.73 ± 0.41
18:3n3	ND	0.23 ± 0.04

**Table 4:** Food intake and body weight of rabbits fed experimental diets in week 0 and 8.

Group	Animal weight (kg/rabbit)		Daily food intake (g/rabbit)	
	Week		Week	
	0	8	0	8
NC	1.87 ± 0.03	2.09 ± 0.06^ab^	79.80 ± 61.58	93.75 ± 2.05^abc^
PC	1.82 ± 0.09	2.34 ± 0.04^b^	80.00 ± 5.44	90.33 ± 2.69^ab^
HS	1.86 ± 0.02	2.22 ± 0.09^ab^	76.71 ± 2.57	82.79 ± 1.52^a^
HPO	1.74 ± 0.02	2.19 ± 0.10^ab^	74.93 ± 0.47	107.50 ± 5.75^c^
HKO	1.74 ± 0.04	2.13 ± 0.07^ab^	81.92 ± 1.39	103.37 ± 4.29^bc^
HF	1.84 ± 0.08	2.02 ± 0.01^a^	75.47 ± 0.27	102.76 ± 5.92^bc^
HD	1.75 ± 0.00	2.24 ± 0.01^ab^	80.49 ± 4.55	107.07 ± 1.14^c^

Values are expressed as mean ± SEM. Values are not significantly different at *P* < 0.05 in week 0. NC: normal diet group, PC: positive control, HS, HPO, HKO, HF, and HD: hypercholesterolemic group treated with 10 mg/kg/day simvastatin, 20 g kg^-1^ of pulp oil, 20 g kg^-1^ kernel oil, 50 g kg^-1^ fullfat pulp, or 50 g kg^-1^ defatted pulp, respectively.

**Table 5:** Plasma lipid profile and the ratio of TC to HDL-C at week 0 (baseline), week 4, and week 8.

	NC	PC	HS	HPO	HKO	HF	HD
TC (mmol/L)							
Week 0	1.79 ± 0.06^a,1^	1.54 ± 0.06^a,1^	1.58 ± 0.09^a,1^	1.16 ± 0.03^a,1^	0.84 ± 0.03^a,1^	1.23 ± 0.01^a,1^	0.89 ± 0.02^a,1^
Week 4	2.21 ± 0.04^a,2^	19.58 ± 0.81^d,2^	9.54 ± 0.38^b,2^	20.4 ± 0.37^d,2^	21.36 ± 0.59^d,2^	12.33 ± 0.47^c,2^	4.21 ± 0.19^a,2^
Week 8	1.94 ± 0.02^a,1^	34.42 ± 0.84^c,3^	3.74 ± 0.11^a,3^	24.83 ± 0.88^b,3^	24.26 ± 0.61^b,3^	33.61 ± 0.99^c,3^	1.29 ± 0.02^a,1^

LDL-C (mmol/L)							
Week 0	0.66 ± 0.03^a,1^	0.62 ± 0.03^a,1^	0.65 ± 0.02^a,1^	0.53 ± 0.21^a,1^	0.28 ± 0.18^a,1^	0.58 ± 0.04^a,1^	0.29 ± 0.02^a,1^
Week 4	1.02 ± 0.05^a,2^	11.94 ± 0.06^c,2^	5.97 ± 0.12^b,2^	13.17 ± 0.89^c,2^	6.36 ± 0.42^c,2^	14.91 ± 0.61^b,2^	3.00 ± 0.22^ab,2^
Week 8	0.66 ± 0.05^a,1^	14.84 ± 1.26^c,2^	3.99 ± 0.31^a,3^	14.83 ± 1.42^b,2^	17.37 ± 1.14^c,3^	17.97 ± 1.18^c,2^	10.90 ± 0.59^b,3^

HDL-C (mmol/L)							
Week 0	0.59 ± 0.04^c,1^	0.36 ± 0.02^ab,1^	0.49 ± 0.03^bc,1^	0.37 ± 0.03^ab,1^	0.35 ± 0.02^ab,1^	0.55 ± 0.04^c,1^	0.30 ± 0.03^a,1^
Week 4	0.6 ± 0.04^a,1^	2.81 ± 0.11^c,2^	2.46 ± 0.03^c,2^	4.08 ± 0.38^d,2^	1.59 ± 0.07^c,2^	2.62 ± 0.22^b,2^	0.68 ± 0.16^a,1^
Week 8	0.57 ± 0.04^a,1^	3.59 ± 0.19^d,3^	1.29 ± 0.13^a,3^	2.79 ± 0.21^c,3^	3.31 ± 0.11^d,3^	4.12 ± 0.24^e,3^	1.91 ± 0.11^b,2^

TG (mmol/L)							
Week 0	0.64 ± 0.01^a,1^	0.65 ± 0.06^a,1^	0.56 ± 0.02^a,1^	0.69 ± 0.05^a,1^	0.65 ± 0.03^a,1^	0.69 ± 0.05^a,1^	0.75 ± 0.11^a,1^
Week 4	0.54 ± 0.01^c,2^	0.48 ± 0.03^ab,1^	0.59 ± 0.12^a,1^	0.7 ± 0.05^a,1^	0.77 ± 0.07^a,1^	0.43 ± 0.03^b,1^	1.07 ± 0.08^d,1^
Week 8	0.51 ± 0.04^a,1^	1.28 ± 0.09^c,2^	0.56 ± 0.04^a,1^	1.28 ± 0.05^c,2^	1.58 ± 0.07^c,1^	1.18 ± 0.12^b,2^	1.03 ± 0.02^c,1^

TC : HDL ratio							
Week 0	2.99 ± 0.01^b,1^	4.29 ± 0.11^d,1^	3.22 ± 0.08^b,2^	3.13 ± 0.05^b,1^	3.50 ± 0.09^c,2^	1.53 ± 0.06^a,1^	2.91 ± 0.14^b,2^
Week 4	3.68 ± 0.01^a,2^	6.98 ± 0.19^a,2^	3.88 ± 0.07^a,3^	5.01 ± 0.11^b,2^	7.74 ± 0.50^d,3^	8.14 ± 0.70^e,3^	6.22 ± 0.15^c,3^
Week 8	3.39 ± 0.08^b,3^	9.57 ± 0.11^b,3^	1.36 ± 0.14^a,1^	8.88 ± 0.10^d,3^	10.15 ± 0.42^e,1^	5.89 ± 0.09^c,2^	0.67 ± 0.02^a,1^

Value represents the mean ± SEM (*n* = 4). Values for a given parameter in a row that do not share the same superscript letter are significantly different at *P* < 0.05. Values for a given parameter in each column that do not share the same superscript number are significantly different at *P* < 0.05. NC: normal control, PC: positive control, HS, HPO, HKO, HF, and HD: hypercholesterolemic diet treated with simvastatin, 20 g kg^−1^ of pulp oil, 20 g kg^−1^ kernel oil, 50 g kg^−1^ fullfat pulp, or 50 g kg^−1^ defatted pulp, respectively.

**Table 6:** Plasma malondialdehyde (MDA) level in experimental rabbits.

Groups	Plasma MDA (mol/L)		
	Week 0	Week 4	Week 8
NC	2.60 ± 0.04^a,1^	2.67 ± 0.08^b,2^	3.74 ± 0.15^b,2^
PC	2.92 ± 0.32^a,1^	3.10 ± 0.17^ab,1^	4.37 ± 0.18^c,2^
HS (simvastatin)	2.80 ± 0.10^a,1^	2.87 ± 0.07^c,1^	4.17 ± 0.17^bc,2^
HPO (pulp oil)	2.96 ± 0.08^a,1^	3.42 ± 0.03^b,2^	3.78 ± 0.08^b,3^
HKO (kernel oil)	3.03 ± 0.18^a,1^	3.44 ± 0.15^b,1^	3.79 ± 0.05^b,2^
HF (fullfat)	3.06 ± 0.12^a,1^	3.06 ± 0.11^b,1^	3.70 ± 0.09^b,2^
HD (defatted)	2.92 ± 0.18^a,1^	3.77 ± 0.09^a,2^	2.81 ± 0.04^a,1^

NC: normal diet group, PC: positive control, HS, HPO, HKO, HF, and HD: hypercholesterolemic group treated with 10 mg/kg/day simvastatin, 20 g kg^−1^ of pulp oil, 20 g kg^−1^ kernel oil, 50 g kg^−1^ fullfat pulp, or 50 g kg^−1^ defatted pulp, respectively. Values are expressed as mean ± SEM. Values within column followed by the same superscript letter are not significantly different at *P* > 0.05. Values within groups followed by the same superscript number are not significantly different at *P* > 0.05.

## References

[1] Heistad DD, Wakisaka Y, Miller J, Chu Y, Pena-Silva R (2009). Novel aspects of oxidative stress in cardiovascular diseases. *Circulation Journal*.

[2] Prasad K (2005). Hypocholesterolemic and antiatherosclerotic effect of flax lignan complex isolated from flaxseed. *Atherosclerosis*.

[3] Toshima SI, Hasegawa A, Kurabayashi M (2000). Circulating oxidized low density lipoprotein levels: a biochemical risk marker for coronary heart disease. *Arteriosclerosis, Thrombosis, and Vascular Biology*.

[4] Barter PJ, Nicholls S, Rye KA, Anantharamaiah GM, Navab M, Fogelman AM (2004). Antiinflammatory properties of HDL. *Circulation Research*.

[5] Assmann G, Gotto AM (2004). HDL cholesterol and protective factors in atherosclerosis. *Circulation*.

[6] Barter P, Kastelein J, Nunn A (2003). High density lipoproteins (HDLs) and atherosclerosis; the unanswered questions. *Atherosclerosis*.

[7] Albertini R, Moratti R, De Luca G (2002). Oxidation of low-density lipoprotein in atherosclerosis from basic biochemistry to clinical studies. *Current Molecular Medicine*.

[8] Bonetti PO, Lerman LO, Lerman A (2003). Endothelial dysfunction: a marker of atherosclerotic risk. *Arteriosclerosis, Thrombosis, and Vascular Biology*.

[9] Mates JM, Rerez-Gomez C, Castro IN (1999). Antioxidant enzymes and human disease. *Clinical Biochemistry*.

[10] Chai CC, Teo GK, Lau CY, Powoven AMA (2008). Conservation and sustinable utilization of indigenous vegetables of Sarawak. *Agrobiodiversity in Malaysia*.

[11] Wong WWW, Chong TC, Tananak J (2008). Conservation and sustainable utilization of fruit species of Sabah. *Agrobiodiversity in Malaysia*.

[12] Ding P, Tee YK (2011). Physicochemical characteristics of dabai (*Canarium odontophyllum* Miq.) fruit. *Fruits*.

[13] Hoe VB (1999). The nutritional value of indigenous fruits and vegetables in Sarawak. *Asia Pacific Journal of Clinical Nutrition*.

[14] Hierro MTG, Tomas MC, Fernandez-Martin F, Santa-Maria G (1992). Determination of the triglyceride composition of avocado oil by high-performance liquid chromatography using a light-scattering detector. *Journal of Chromatography*.

[15] Salvador MD, Aranda F, Fregapane G (2001). Influence of fruit ripening on ’Cornicabra’ virgin olive oil quality: a study of four successive crop seasons. *Food Chemistry*.

[16] Shakirin FH, Prasad KN, Ismail A, Yuon LC, Azlan A (2010). Antioxidant capacity of underutilized Malaysian *Canarium odontophyllum* (dabai) Miq. fruit. *Journal of Food Composition and Analysis*.

[17] Azrina A, Nurul Nadiah MN, Amin I (2010). Antioxidant properties of methanolic extract of *Canarium odontophyllum* fruit. *International Food Research Journal*.

[18] Chew LY, Khoo HE, Amin I, Azrina A, Lau CY (2011). Analysis of phenolic compounds of dabai (*Canarium odontophyllum* Miq.) fruits by high-performance liquid chromatography. *Food Analytical Methods*.

[19] Hainida E, Ismail A, Hashim N, Mohd N, Zakiah A (2008). Effects of defatted dried roselle (*Hibiscus sabdariffa* L.) seed powder on lipid profiles of hypercholesterolemia rats. *Journal of the Science of Food and Agriculture*.

[20] Lecumberri E, Goya L, Mateos R (2007). A diet rich in dietary fiber from cocoa improves lipid profile and reduces malondialdehyde in hypercholesterolemic rats. *Nutrition*.

[21] Buege JA, Aust SD (1978). Microsomal lipid peroxidation. *Methods in Enzymology*.

[22] Holman RL, McGill HC, Strong JP, Geer JC (1958). The natural history of atherosclerosis: the early aortic lesions as seen in New Orleans in the middle of the of the 20th century. *The American Journal of Pathology*.

[23] Bocan TMA, Mueller SB, Mazur MJ, Uhlendorf PD, Brown EQ, Kieft KA (1993). The relationship between the degree of dietary-induced hypercholesterolemia in the rabbit and atherosclerotic lesion formation. *Atherosclerosis*.

[24] Georges AN, Olivier CK, Simard RE (1992). *Canarium schweinfurthii* Engl.: chemical composition of the fruit pulp. *Journal of the American Oil Chemists’ Society*.

[25] Chew LY, Prasad KN, Amin I, Azrina A, Lau CY (2011). Nutritional composition and antioxidant properties of *Canarium odontophyllum* Miq. (dabai) fruits. *Journal of Food Composition and Analysis*.

[26] Jimenez A, Rodriguez R, Fernandez-Caro I, Guilen R, Fernandez-Bolanos J, Heredia A (2000). Dietary fibre content of tables olives processed under different European styles: study of physico-chemical characteristics. *Journal of the Science of Food and Agriculture*.

[27] Gorinstein S, Caspi A, Libman I, Katrich E, Lerner HT, Trakhtenberg S (2004). Preventive effects of diets supplemented with sweetie fruits in hypercholesterolemic patients suffering from coronary artery disease. *Preventive Medicine*.

[28] Zunft HJF, Lüder W, Harde A (2003). Carob pulp preparation rich in insoluble fibre lowers total and LDL cholesterol in hypercholesterolemic patients. *European Journal of Nutrition*.

[29] Sudha ML, Baskaran V, Leelavathi K (2007). Apple pomace as a source of dietary fiber and polyphenols and its effect on the rheological characteristics and cake making. *Food Chemistry*.

[30] Nurnadia A (2007). *The Determination of Fatty Acid Composition and Vitamin E Content in Oils Extracted from Flesh and Kernel of Canarium odontophyllum*.

[31] Chen CC, Liu LK, Hsu JD, Huang HP, Yang MY, Wang CJ (2005). Mulberry extract inhibits the development of atherosclerosis in cholesterol-fed rabbits. *Food Chemistry*.

[32] Lee HS, Ahn HC, Ku SK (2006). Hypolipemic effect of water extracts of picrorrhiza rhizoma in PX-407 induced hyperlipemic ICR mouse model with hepatoprotective effects: a prevention study. *Journal of Ethnopharmacology*.

[33] Weintraub H Simvastatin 80mg: if you can't go lower, go elsewhere.

[34] Hu FB, Willett WC (2002). Optimal diets for prevention of coronary heart disease. *Journal of the American Medical Association*.

[35] Xing WW, Wu JZ, Jia M, Du J, Zhang H, Qin LP (2009). Effects of polydatin from *Polygonum cuspidatum* on lipid profile in hyperlipidemic rabbits. *Biomedicine and Pharmacotherapy*.

[36] Garg ML, Blake RJ, Wills RBH (2003). Macadamia nut consumption lowers plasma total and LDL cholesterol levels in hypercholesterolemic men. *Journal of Nutrition*.

[37] Rauch U, Osende JI, Chesebro JH (2000). Statins and cardiovascular diseases: the multiple effects of lipid-lowering therapy by statins. *Atherosclerosis*.

[38] Johnston TP, Nguyen LB, Chu WA, Shefer S (2001). Potency of select statin drugs in a new mouse model of hyperlipidemia and atherosclerosis. *International Journal of Pharmaceutics*.

[39] Rosenson RS (2004). Statins in atherosclerosis: lipid-lowering agents with antioxidant capabilities. *Atherosclerosis*.

[40] Khoo HE, Azlan A, Ismail A, Abas F (2012). Influence of different extraction media on phenolic contents and antioxidant capacity of defatted dabai (*Canarium odontophyllum*) fruit. *Food Analytical Methods*.

[41] Prasad KN, Chew LY, Khoo HE, Yang B, Azlan A, Ismail A (2011). Carotenoids and antioxidant capacities from *Canarium odontophyllum* Miq. fruit. *Food Chemistry*.

[42] Kerckhoffs DAJM, Hornstra G, Mensink RP (2003). Cholesterol-lowering effect of *β*-glucan from oat bran in mildly hypercholesterolemic subjects may decrease when *β*-glucan is incorporated into bread and cookies. *American Journal of Clinical Nutrition*.

[43] Kris-Etherton PM, Hecker KD, Bonanome A (2002). Bioactive compounds in foods: their role in the prevention of cardiovascular disease and cancer. *American Journal of Medicine*.

[44] Kang MH, Kawai Y, Naito M, Osawa T (1999). Dietary defatted sesame flour decreases susceptibility to oxidative stress in hypercholesterolemic rabbits. *Journal of Nutrition*.

[45] Queenan KM, Stewart ML, Smith KN, Thomas W, Fulcher RG, Slavin JL (2007). Concentrated oat *β*-glucan, a fermentable fiber, lowers serum cholesterol in hypercholesterolemic adults in a randomized controlled trial. *Nutrition Journal*.

[46] Chu WW, Hanson PG (2000). Dietary fiber and coronary artery disease. *Wisconsin Medical Journal*.

[47] Ramos S, Moulay L, Granado-Serrano AB (2008). Hypolipidemic effect in cholesterol-fed rats of a soluble fiber-rich product obtained from cocoa husks. *Journal of Agricultural and Food Chemistry*.

[48] Anderson JW, Baird P, Davis RH (2009). Health benefits of dietary fiber. *Nutrition Reviews*.

[49] Yuan G, Al-Shali KZ, Hegele RA (2007). Hypertriglyceridemia: its etiology, effects and treatment. *Canadian Medical Association Journal*.

[50] Leudeu BCT, Tchiegang C, Gadet MD (2006). Effect of *Canarium Scheweinfurthii *and *Dacrydodes edulis* oils on blood lipids, lipid peroxidation and oxidative stress in rats. *Journal of Food Technology*.

[51] Mensink RP, Zock PL, Kester ADM, Katan MB (2003). Effects of dietary fatty acids and carbohydrates on the ratio of serum total to HDL cholesterol and on serum lipids and apolipoproteins: a meta-analysis of 60 controlled trials. *American Journal of Clinical Nutrition*.

[52] Moreno JJ, Mitjavila MT (2003). The degree of unsaturation of dietary fatty acids and the development of atherosclerosis. *Journal of Nutritional Biochemistry*.

[53] Stocker R, Keaney JF (2004). Role of oxidative modifications in atherosclerosis. *Physiological Reviews*.

[54] Amom Z, Zakaria Z, Mohamed J (2008). Lipid lowering effect of antioxidant alpha-lipoic acid in experimental atherosclerosis. *Journal of Clinical Biochemistry and Nutrition*.

[55] Prasad K, Mantha SV, Muir AD, Westcott ND (1998). Reduction of hypercholesterolemic atherosclerosis by CDC-flaxseed with very low alpha-linolenic acid. *Atherosclerosis*.

[56] Triviño A, Ramírez AI, Salazar JJ (2006). A cholesterol-enriched diet induces ultrastructural changes in retinal and macroglial rabbit cells. *Experimental Eye Research*.

[57] Hakimoğlu F, Kizil G, Kanay Z, Kizil M, Isi H (2007). The effect of ethanol extract of Hypericum lysimachioides on lipid profile in hypercholesterolemic rabbits and its *in vitro* antioxidant activity. *Atherosclerosis*.

[58] Gonçalves S, Maria AV, Silva-Herdade AS, Martins e Silva J, Saldanha C (2007). Milk enriched with phytosterols reduces plasma cholesterol levels in healthy and hypercholesterolemic subjects. *Nutrition Research*.

[59] Prasad K (2010). Effects of vitamin e on serum enzymes and electrolytes in hypercholesterolemia. *Molecular and Cellular Biochemistry*.

[60] Schwenke DC, Behr SR (1998). Vitamin E combined with selenium inhibits atherosclerosis in hypercholesterolemic rabbits independently of effects on plasma cholesterol concentrations. *Circulation Research*.

